# Individual patient data meta-analysis of acupuncture for chronic pain: protocol of the Acupuncture Trialists' Collaboration

**DOI:** 10.1186/1745-6215-11-90

**Published:** 2010-09-28

**Authors:** Andrew J Vickers, Angel M Cronin, Alexandra C Maschino, George Lewith, Hugh Macpherson, Norbert Victor, Karen J Sherman, Claudia Witt, Klaus Linde

**Affiliations:** 1 Department of Epidemiology and Biostatistics, Memorial Sloan-Kettering Cancer Center, New York, NY 10065, USA; 2 Center for Outcomes and Policy Research, Department of Medical Oncology, Dana-Farber Cancer Institute, 44 Binney St, Boston, Massachusetts 02115, USA; 3 Complementary and Integrated Medicine Research Unit, Southampton Medical School, Southampton, UK; 4 Complementary Medicine Research Group, University of York, York, UK; 5 Department of Medical Biometry and Informatics, University of Heidelberg, Heidelberg, Germany; 6 Group Health Research Institute, Group Health Cooperative, Seattle, WA, USA; 7 Institute for Social Medicine, Epidemiology, and Health Economics, Charité, University Medical Center, Berlin, Germany; 8 Institute of General Practice, Technische Universität München, Munich, Germany

## Abstract

**Background:**

The purpose of clinical trials of acupuncture is to help clinicians and patients make decisions about treatment. Yet this is not straightforward: some trials report acupuncture to be superior to sham (placebo) acupuncture while others show evidence that acupuncture is superior to usual care but not sham, and still others conclude that acupuncture is no better than usual care. Meta-analyses of these trials tend to come to somewhat indeterminate conclusions. This appears to be because, until recently, acupuncture research was dominated by small trials of questionable quality. The Acupuncture Trialists' Collaboration, a group of trialists, statisticians and other researchers, was established to synthesize patient-level data from several recently published large, high-quality trials.

**Methods:**

There are three distinct phases to the Acupuncture Trialists Collaboration: a systematic review to identify eligible studies; collation and harmonization of raw data; statistical analysis. To be eligible, trials must have unambiguous allocation concealment. Eligible pain conditions are osteoarthritis; chronic headache (tension or migraine headache); shoulder pain; and non-specific back or neck pain. Once received, patient-level data will undergo quality checks and the results of prior publications will be replicated. The primary analysis will be to determine the effect size of acupuncture. Each trial will be evaluated by analysis of covariance with the principal endpoint as the dependent variable and, as covariates, the baseline score for the principal endpoint and the variables used to stratify randomization. The effect size for acupuncture from each trial - that is, the coefficient and standard error from the analysis of covariance - will then be entered into a meta-analysis. We will compute effect sizes separately for comparisons of acupuncture with sham acupuncture, and acupuncture with no acupuncture control for each pain condition. Other analyses will investigate the impact of different sham techniques, styles of acupuncture or frequency and duration of treatment sessions.

**Discussion:**

Individual patient data meta-analysis of high-quality trials will provide the most reliable basis for treatment decisions about acupuncture. Above all, however, we hope that our approach can serve as a model for future studies in acupuncture and other complementary therapies.

## Background

### Introduction

The term "acupuncture" is used to describe a heterogeneous set of healthcare practices, with a spectrum of styles from "traditional" acupuncture through to "Western" acupuncture. Common to these styles of acupuncture is the insertion and stimulation of needles at specific points on the body to facilitate recovery of health.

Chronic pain is the most common presenting condition for acupuncturists in the Western world [1,2]. Pain has also be the focus of most clinical research on acupuncture. In June 2010, MEDLINE listed slightly over 1100 English-language randomized trials for acupuncture and approximately half of these concerned pain. The clear and obvious purpose of such trials is to inform clinical practice. However, using acupuncture trials to aid clinical decisions is not straightforward. To use a simple example, imagine that a patient with chronic low back pain consulted with a physician to determine whether acupuncture might be an appropriate treatment. The physician is unsure of the evidence and so searches MEDLINE using the terms 'acupuncture' and 'back pain' with 'randomized controlled trial' publication type as a limit. This retrieves 74 different papers, some of which suggest that acupuncture is superior to both sham (placebo) acupuncture and no acupuncture control[3]; others that report acupuncture to be superior to no acupuncture but not to sham acupuncture[4] and yet others failing to find differences between acupuncture and no acupuncture[5]. This illustrates the need for studies that synthesize existing research.

There have been a large number of systematic reviews on acupuncture for pain. For example, the 2nd issue of the Cochrane Library for 2007 listed 12 Cochrane Collaboration reviews of acupuncture for pain, with over 50 additional non-Cochrane systematic reviews published on this topic. As an example of an acupuncture systematic review, consider Ezzo et al.'s paper on acupuncture for the treatment of chronic pain, published in 2000[6]. This included 51 randomized trials of acupuncture for a variety of conditions, including back pain, headache, osteoarthritis and dysmenorrhea. The study can be seen as typical of acupuncture systematic reviews for three reasons. First, the sample size of the included studies was low: there were a total of 2423 patients in the 51 analyzed trials, with a median sample size per group of 18. Second, the methodological quality of the studies was questionable: 68% of the studies in the review were defined as poor quality and only 3 of the 51 studies received a maximum quality score. Third, and as a consequence of the first two points, the conclusions drawn by the reviewers are very tentative: "there is limited evidence that acupuncture is more effective than no treatment for chronic pain; and inconclusive evidence that acupuncture is more effective than placebo".

The landscape of clinical research in acupuncture has recently been dramatically altered by the completion of several large, high quality trials. The first of these to be published was a trial of acupuncture for chronic headache disorders: the trial randomized 401 patients for acupuncture or usual care control[7] and, at the time, was the largest randomized trial for acupuncture for pain conducted in the West; however, it was quickly superseded by Berman et al.'s trial of acupuncture for osteoarthritis of the knee (n = 570)[8]. The German ART (Acupuncture Randomized Trials) studies published in 2005 and early 2006[4,9-11] included close to 300 patients each in four separate trials on osteoarthritis, chronic low back pain, migraine and chronic tension headache. But even these trials are dwarfed by the GERAC (GERman ACupuncture) trials which accrued close to 1000 patients each on trials on osteoarthritis[12], chronic low back pain[13] and migraine[14] and 400 on chronic tension headache[15]. The ARC (Acupuncture in Routine Care) trials have even larger sample sizes: 3000 patients or more on each three separate trials of back pain[16], neck pain[17] and chronic headache[18], and 700 patients on a trial of arthritis[19].

We suggest that the optimal method to synthesize existing acupuncture trials is to conduct individual patient data meta-analysis, including only the highest quality trials. The Acupuncture Trialists' Collaboration was established to obtain raw data from investigators and combine these into a single data set[20]. This data set will be analyzed to address questions concerning both the management of chronic pain conditions and research design for acupuncture.

### Objectives

Our overarching objective is:

**1. To establish an individual patient level database of raw data from high quality trials of acupuncture for chronic pain conditions**. We will analyze this database to address several key hypotheses in acupuncture research.

Our primary analytic objectives are:

**2. To determine whether real acupuncture is superior to sham acupuncture for the treatment of chronic pain conditions and, if so, to determine the effect size**. Here 'real acupuncture' is defined as the experimental intervention which the investigators believe may have activity against pain. 'Sham acupuncture' is defined as any intervention that is designed to prevent the patient from knowing whether or not he or she received real acupuncture but which is thought to have minimal activity against pain.

**3. To determine whether real acupuncture is superior to no acupuncture control for the treatment of chronic pain conditions and, if so, to determine the effect size**. 'No acupuncture control' is defined to include care, such as medication 'as needed', that is also received by the acupuncture group. No acupuncture controls are sometimes described as 'waiting list' controls, 'usual' or 'standard care' controls or controls receiving 'no additional treatment'. 'Attention control', where patients receive general education and advice, are also included in this category.

For objectives 2 and 3, analyses will be conducted separately for each pain condition (musculoskeletal, osteoarthritis, headache) and then within pain condition (neck pain, shoulder pain, back pain; chronic tension headache, migraine). Our secondary objectives are:

**1. To determine the effect size of acupuncture for pain, function, health related quality of life and medication use**. These analyses will be conducted separately for the different pain conditions and comparators, and at different follow-up times.

**2. To determine which aspects of acupuncture treatment or sham control affect outcome**. These analyses include questions such as whether effects vary by the indication or the style of acupuncture, or whether different practitioners have different outcomes.

## Methods/Design

The study is exempt from IRB oversight because it involves analysis of de-identified data that has already been collected for a separate purpose.

### Phase I: Systematic review to identify eligible papers

#### Trial eligibility criteria

##### Methodology

It has been demonstrated that unconcealed allocation is the most important source of bias in randomized trials[21,22]. We will therefore include only those randomized trials of acupuncture for chronic pain conditions where allocation concealment is determined unambiguously to be adequate. In cases where this is not clear from the published paper, we will contact authors for further information concerning the exact logistics of the randomization process.

The Acupuncture Trialists' Collaboration will only include trials with adequate allocation concealment. We consider allocation to be adequately concealed if both of the following two conditions hold:

1. Researchers were unable to predict the group to which a patient would be randomized until the patient was unambiguously registered on study.

2. Researchers were unable to change a patient's allocation after a patient was randomized.

Examples for adequate concealment of allocation are given in table 1. Allocation concealment will be considered inadequate if participants or investigators enrolling participants could possibly foresee or modify assignments and thus introduce selection bias. Table 1 provides some examples of inadequate allocation concealment.

**Table 1:** Examples of adequate and inadequate allocation concealment

Adequate allocation concealment	Inadequate allocation concealment
Centralized randomization procedures in which the persons including a patient contact the randomization center or a person otherwise not involved in the trial who registers the patient as included in the trial and only then provides the allocation information for this patient	An open random allocation schedule, that is, where all future allocations can be read by an investigator

Any computerized system that ensures, by password protection or other computer security procedures, conditions 1 and 2 described in the text	Envelopes, if clear details of the procedures used to avoid allocation becoming unconcealed are inadequate, or unclear

Use of consecutively numbered, opaque sealed envelopes containing the allocation information kept by a person otherwise not involved in the study with envelopes opened only after registration of an included patient	Assignment envelopes were used without appropriate safeguards (for example if envelopes were unsealed or non-opaque or not sequentially numbered)

	Alternation, rotation, day of the week

	Date of birth, or medical record number

	Blocked randomization with block size known to the person including the patient

	An unspecified method, for example, if the report states only that "patients were randomized" without giving details of the exact procedures used, and no further information is obtained from the authors.

In the case of envelope randomization, investigators must establish clear procedures to ensure conditions 1 and 2 above. For example, there should be procedures to prevent investigators resealing and reusing an envelope after it was opened (e.g. envelopes are held by an independent party); reading the contents (e.g. use of cardboard or silver foil) or selecting multiple envelopes (e.g. sequential numbering).

##### Patients

Eligible pain conditions are osteoarthritis; chronic or recurrent headaches such as tension or migraine headache; specific and non-specific shoulder pain; and non-specific back or neck pain. Trials of back or neck pain associated with specific pathologies (e.g. osteoporotic fracture) will be excluded. Trials of shoulder pain associated with specific pathologies (e.g. rotator cuff tendonitis, frozen shoulder, or bursitis) will be included. Our rationale is that our main analyses will be conducted separately by indication, and for indications other than those listed, we do not believe that we will identify more than 1 or 2 eligible trials. For osteoarthritis and headache pain we will not require a specific pain duration, as both are chronic in nature. Back, neck and shoulder pain are episodic conditions and we will use the criterion used in many trials that the current episode must be of at least four weeks duration. In a normal systematic review, summary data are analyzed and so a trial is included only if all patients meet a criterion. In an individual patient data meta-analysis, such as this, we have the possibility of including a trial, but excluding certain patients on that trial from analysis.

a) If a trialist can provide data on duration of symptoms, the trial is eligible. Only patients with a duration of 4 weeks or more are included in the analysis.

b) If a trialist does not provide data on duration of symptoms, duration of 4 weeks or more must be an eligibility criterion.

##### Interventions

Trials where acupuncture points or trigger points are stimulated by acupuncture needles. Trials will be classed as ineligible if patients in the acupuncture group, but not the control group, were protocolled to receive medication (conventional or otherwise), surgery or physical therapy.

##### Controls

At least one group in the trial must receive sham acupuncture or no acupuncture control.

*Sham acupuncture *is defined as any intervention that is designed to prevent the patient from knowing whether or not he or she received real acupuncture but which is thought to have minimal activity against pain. This includes superficial needle insertion; needle insertion at non-acupuncture points or at points not indicated for the condition under study; 'placebo' needles, such as the Streitberger needle[23], which appear to penetrate the skin but which do not do so; techniques, such as tapping on a guide-tube, designed to feel like needle penetration; and non-needle methods such as detuned lasers or deactivated transcutaneous electric nerve stimulation devices. It is worth noting that we do not consider these controls to be equivalent *a priori; *possible differences between sham procedures will be analyzed as one of our objectives.

*No acupuncture control *is defined as any of the following apply: 1) Care received in the control group is also available to the acupuncture group. Examples include a trial with waiting list control, one in which patients receive usual clinical care with or without acupuncture and a study where the effects of a course of physiotherapy plus acupuncture are compared to physiotherapy alone. This category may also include studies where care in the acupuncture and control groups is not identical, but does not differ in any medically substantive manner. 2) The intervention in the control group involves general advice, education and support. This type of control is sometimes described as 'attention control'. 3) The control group, but not the acupuncture group, received recommendations for guideline care, although no specific treatment plan was mandated and no treatment was provided by the trial. As is the case for sham acupuncture, we do not expect that these different types of no acupuncture control will have equivalent effects, but we will include different types of control in our analyses and investigate differences between them. Control groups will be excluded if, in addition to sham acupuncture or treatments also available in the true acupuncture group, controls received a specific program of treatment such as medication, massage or physical therapy.

##### Outcomes

There is no restriction on eligibility due to the type of endpoint. However, on the grounds that we are interested in the effects of acupuncture on medium and long-term pain, the primary endpoint must be measured more than four weeks after the initial acupuncture treatment.

##### Trial size

There will be no restrictions on the size of the trial. However, for expediency, we will only invite as collaborators trialists associated with trials of 100 or more patients. Trialists of eligible studies with fewer than 100 patients will be invited to submit raw data to the collaboration but not to join the collaboration as a member.

##### Language

There are no language exclusions. All papers in languages other than English will be translated into English and the English text made available to all collaborators.

#### Search strategy for identification of studies

We will search MEDLINE, CENTRAL (the Cochrane Collaboration Database) and the citation lists of systematic reviews. The search strategy used will be as for the prior reviews of headache[24], back pain[25] and osteoarthritis[26] (each of which was co-authored by one or more members of the Acupuncture Trialists Collaboration) with the addition of the following terms: neck, shoulder, cervical, musculoskeletal.

Searching established databases for trials conducted in China or published in the Chinese language is likely to have very poor precision as very few of these studies are of sufficient quality to merit inclusion in the Acupuncture Trialists' Collaboration[27-30]. Accordingly, trials identified by the standard searches (e.g. of Medline) that were conducted in China will not be considered further. Chinese trials will be identified by a separate process: Jianping Liu of the Chinese Cochrane Center will use that institution's resources to identify trials of acupuncture for chronic pain that involved full allocation concealment.

#### Inclusion of studies

All retrieved references will be scanned by one of two investigators to remove any clearly inappropriate titles. Hard copies of all remaining papers will then be obtained and read by both investigators to remove any for which there is no possibility of eligibility. Inclusion criteria for the remaining papers will be applied by two reviewers separately (no reviewer may assess a trial on which he or she is listed as a co-author). Disagreements about study inclusion will be resolved by consensus. Authors of trials will be contacted, if necessary, to clarify details such as allocation concealment, if this is not clear. However, authors will not be contacted if a trial has already been included in a prior published systematic review conducted by one of the collaborators: methodological details will be taken from the published review. All retrieved trials excluded from the review will be given reasons for exclusion as follows: not a randomized trial; allocation unclear or inadequate; not acupuncture; inappropriate control; not pain; only short-term measurement of pain; not an osteoarthritis, headache, back, neck or shoulder pain trial.

#### Quality assessment

##### Methodological quality

The most important quality criteria for a randomized trial concern the quality of randomization, blinding, and exclusions and drop-outs[21,22]. The quality of randomization is an inclusion criterion for this study: only trials with full allocation concealment will be analyzed. Exclusions and drop-outs will be dealt with by multiple imputation in the statistical analysis. Hence our quality assessment will focus on blinding. For all studies involving sham acupuncture, assessment of blinding will follow prior Cochrane reviews in grading as A, B or C.

A: Low likelihood of bias: EITHER the adequacy of blinding was checked by direct questioning of patients, for example, with a credibility questionnaire, and no important differences were found between groups OR a blinding method (e.g. the Streitberger sham device) was used that had previously been validated as able to maintain blinding.

C: High likelihood of bias: Clear reasons to believe that blinding was broken, for example, differential responses to a credibility questionnaire or obviously non-credible sham technique

B: Intermediate likelihood of bias: A trial that does not meet the criteria for either a grade of A or C.

Note that the results of credibility questionnaires given several weeks after treatment starts are of questionable value as they are confounded by treatment effects: a patient who experienced a rapid improvement in symptoms after treatment would be more likely to believe that he received real acupuncture than a patient who did not benefit. Hence such questionnaires will not be given prominence in the assessment of blinding. Quality assessment will be conducted by two reviewers separately with disagreements resolved by consensus.

##### Adequacy of acupuncture treatment

In addition to assessing the quality of the trials, we will assess the quality of the acupuncture delivered. The method was developed by Hugh MacPherson, a member of the Acupuncture Trialists' Collaboration, and the originator of the STRICTA guidelines[31]. The criteria to assess the adequacy of the acupuncture delivered are:

1.) Fidelity to an adequate protocol OR representative of good practice in the context of what is provided by the same practitioners within their region

2.) Number of sessions

3.) Acupuncturist experience

A panel of expert acupuncturists will be convened. Each panel member will first provide a global assessment for each of the above criteria with three possible ratings: adequate, inadequate and not sufficiently well reported. These ratings will be entered into a data extraction sheet available for review by other panel members. The scores of each rater for each of the three above items will be sent to all panel members. Panel members will then make a judgment about the overall rating of the trial in the same categories of: adequate, inadequate, or not sufficiently well reported to make a judgment. Panel members are recused from assessing any trial on which they are a co-author. Further documentation of the acupuncture techniques used is described under "Data abstraction".

### Phase II: Collection, checking and harmonization of data

#### Development of the database

Individual patient data will be sought for all included trials and entered into a single database. Data will be obtained for all randomized patients, regardless of whether they received treatment or provided post-randomization data. Trial level data will then be added to individual patient records. For example, a data set for a trial might have an indicator variable for acupuncture vs control. This will be replaced by several variables indicating the type of acupuncture and type of control as described in the trial report. Where raw data are not available for a trial, we will conduct sensitivity analyses to determine whether inclusion of the trial might alter our results.

#### Initial data manipulation

The raw data set will be saved in its original format, then converted to a Stata format (Stata is the statistical software used for analysis) and saved again. Three blank statistical programs will then be saved out: one to undertake preliminary checks on the data, one to rename and label the variables and one to replicate statistics reported in the trial publication. All files will be saved using a standard notation: "raw data [descriptor]", "initial import [descriptor]", "initial set up [descriptor]", "initial data checks [descriptor]" and "replication [descriptor]" where "[descriptor]" is a unique label for each data set (e.g. "Linde 2005 migraine").

#### Annotation checks

Statistical code will be written for the "initial set up" program. Each variable in the raw data set will be renamed to a standard notation (e.g. "age_at_randomization" becomes "age") and given a standard label (a label is a text description of the variable, such as "combined headache score at 60 days", that is stored by the statistical software). Variables unique to a particular data set, for example, a WOMAC score in an arthritis trial, will then be identified and labeled. Any variables that cannot be identified, or are ambiguous, will be documented, and appropriate clarification sought from the original investigator.

#### Checking for erroneous or missing data

Statistical code will be written for the "initial data checks" program. First, the number of missing observations for each variable will be calculated and checked against data available in the original publication. Any inconsistencies, or variables for which information on rates of missing data are not available in the trial publication, will be brought to the attention of the original investigator for clarification. Second, "range" checks will be conducted on all variables to determine whether all values are reasonable. As a trivial example, a VAS of 123, or an age of 567 immediately suggest an error. Thirdly, we will check categorical variables by tabulation. For instance, if 200 patients were categorized as having stage I disease, 220 categorized as having stage II disease and 1 as having stage IIa disease, the investigator would be queried as to the accuracy of the IIa categorization.

#### Replication

The third program "replication [descriptor]" will then be written. This will attempt to replicate every number reported on the trial publication. Replications will include baseline characteristics such as age, sex and duration of disease within each group; outcome data such as pain scores within each group at each follow-up time; and comparisons, such as the difference in pain scores between groups at the post-treatment follow-up. In each case, we will use the statistical methods reported by the authors and derive the statistics given in the publication. For example, if a mean and standard deviation for baseline pain score is given in the trial publication, we will similarly calculate mean and standard deviation; if the difference between groups is calculated by linear regression with baseline score and duration of disease as covariates, we will use exactly this method to see if we obtain the same difference between groups, 95% C.I. and p value. Any discrepancies between our results and those reported in the published paper will be brought to the attention of the investigators for clarification. We feel that any data set that has gone through these checks - independent labeling of every variable; assessment of *prima facie *errors; replication of all reported statistics - can be considered valid for inclusion in an independent patient data meta-analysis.

#### Harmonization

Variable names will be harmonized. To indicate time point, variable names will start with "bl" for baseline and "m*j*" or "w*j*" for month *j *and week *j *respectively. Outcome will be categorized as per Additional file 1 and a single tag chosen for each. For example, a visual analog score at 3 months would be named "m3_vas".

### Phase III: Statistical methods

#### Overview

Individual patient data meta-analysis has long been recognized as the ideal method of synthesizing research data. In the words of Iain Chalmers, one of the founders of the Cochrane Collaboration, using individual patient data in a meta-analysis is the "yardstick" by which all meta-analyses should be measured[32]. The advantages of using individual patient data compared to traditional reviews, which analyze published summary data, are as follows[33,34]:

#### Standardization between different analytic approaches

Some trials of acupuncture have reported mean change in pain, others have reported "response rates" of the proportion of patients who experienced a threshold reduction in pain (e.g. 33%). These results cannot be combined without access to raw data, which allows conversion from one type of analysis to another.

#### Application of statistical methods with greater power

In a typical meta-analysis, the investigator records mean and standard deviations for acupuncture and control groups separately. This does not allow the application of techniques, such as analysis of covariance, that have greater statistical power than unadjusted analysis[35,36].

#### Association between patient-level characteristics and outcome

Individual patient data analyses have far greater power to investigate questions such as whether age or baseline symptom severity influence outcome. As an example, if there were four trials with 250 patients each, analysis of published data would attempt to correlate four values of a predictor (e.g. mean age in each trial) with four values of an outcome (e.g. difference between mean pain scores). Analysis of individual patient data would be able to create a model with 1000 data points.

#### Data quality

The process of combining data from different sources requires careful data scrutiny by an independent investigator. This provides an opportunity to identify and correct errors in the data set.

#### Updating trial results

This is an issue of particular importance for trials with survival outcomes as data continue to accrue on a daily basis after publication. This issue may be less pertinent to the Acupuncture Trialists' Collaboration, however, it is possible that trialists have data from long-term follow-up that has yet to be published.

**Figure 1:**
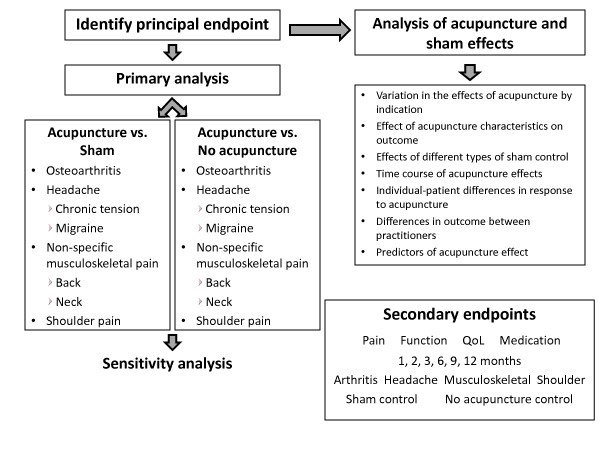
**Overview of the analyses to be conducted**.

On overview of the analyses to be conducted are given in Figure 1.

#### Principal endpoint

For each trial, we will first identify the primary outcome defined by the study authors in terms of both the scale (e.g. WOMAC) and time point (e.g. 6 months after randomization). We will keep endpoints on the continuous scale. For example, in some studies, the primary endpoint was defined in terms of the proportion of patients who had at least a 35% reduction in the number of days with headache pain at 6 month follow-up; in this case, the primary endpoint will be number of days with pain at 6 months. If multiple criteria are considered in the primary outcome, or if the primary outcome is inherently categorical, we will use a continuous measure of pain measured at the same time point as the original primary endpoint. For example, if a trial's primary outcome is a response to treatment defined as a given degree of improvement of a pain scale or a function scale, we would select the pain scale for inclusion in our primary analysis. If there are multiple pain measurements we will select one according to the Outcome Measure Preferences (Additional file 1). For analyses that include trials with different primary endpoints, we will create a standardized primary endpoint by dividing by standard deviation.

#### Primary analysis: analysis of the effect size of acupuncture

Each trial will be reanalyzed by analysis of covariance with the standardized principal endpoint as the dependent variable, and baseline principal endpoint and variables used to stratify randomization as covariates. This approach has been shown to have the greatest statistical power for trials in general with baseline and follow-up measures[36] and also specifically applied to acupuncture research[35]. For trials where randomization was stratified by center or practitioner, this stratification will be included in analysis only if there are 20 or fewer sites and there are a mean of at least 20 patients per site, with at least one patient in each arm at each site. In trials where there is more than one acupuncture group, for example, trials in which patients are randomized to local points, distal points or sham points, results from both real acupuncture groups will be combined (local points and distal points in this case). The effect size for acupuncture from each trial (i.e. the coefficient and standard error) will then be entered into a meta-analysis: the meta-analytic statistics are created by weighting each coefficient by the reciprocal of the variance, summing and dividing by the sum of the weights. Meta-analysis will be accomplished using the *metan *command in Stata (Stata Corp., College Station, TX). Our primary analysis will be a fixed effects model. Our rationale is that a fixed effects analysis constitutes a valid test of the null hypothesis of no treatment effect. Moreover, we take the view that the use of a fixed effects model does not imply an assumption that all trials are estimating the same effect, but that the robustness of the fixed effects approach is likely to lead to a more accurate estimate. Nonetheless, we will report the results of the random effects analysis. We will also report heterogeneity statistics[37].

We will compute effect sizes separately for comparisons of acupuncture with sham and no acupuncture control. Comparisons between acupuncture and sham will omit trials graded as C ('high likelihood of bias') for blinding. These analyses will be conducted separately for each pain condition (specific shoulder conditions, musculoskeletal, osteoarthritis, headache) and then within pain condition (neck pain, back pain; chronic tension headache, migraine).

#### Secondary analyses

We will repeat the analyses of effect size for the secondary endpoints of pain intensity, pain frequency, functional impairment, combined measures of pain and functional impairment, mental well-being (e.g. SF-36 mental health), physical well-being (e.g. SF-36 physical health), overall quality of life (e.g. global assessment), range of motion or stiffness, health change, satisfaction with care and medication use. If a trial reports more than one endpoint that can be placed in a particular category, the outcomes measure preference list (Additional file 1) will be consulted to select the most appropriate measure. On occasion this may involve taking a mean score of two endpoints, for example, if a trial reported both a daytime and nighttime VAS score, we would calculate the average for each patient and the combined score would then be entered into analysis. Note that this demonstrates a key advantage of individual patient data meta-analysis: such a data manipulation would not be possible with summary level data. As it likely that different measurement scales will be used in the different trials, we anticipate that we will use standardized mean differences as the meta-analytic statistic.

Time will always be measured from randomization. For our data, we will use endpoints of 1, 2, 3, 6 and 9 months and one year. For outcomes with these exact time points (or the equivalent in another unit of time: 13 weeks = 3 months), no time point standardization is required. Otherwise, the time point closest to the selected scheme is adopted. For example, if there is no measurement at 6 months but there is for 24 weeks, this timepoint will be selected and relabeled appropriately.

Numerical rating scale (NRS) scores will be converted to a 0 - 100 point scale by appropriate multiplication.

#### Sensitivity analyses

The first sensitivity analysis will involve multiple imputation for missing data, following the approach used in the analysis of the NHS acupuncture for headache trial[7]. In brief, results for patients failing to complete data are imputed using statistical models that are based on available data and take into account sampling variation.

The second sensitivity analysis will be for publication bias. Although we do not believe that there will be many unpublished adequately concealed acupuncture trials large enough to have an important weight in the meta-analysis, we will give characteristics of scenarios that could change the study results. For example, if we found a statistically significant difference between acupuncture and sham, we would estimate the parameters for the following scenarios of trials which, if added to the meta-analysis, would change the p value to 0.05: a) the number of trials with 50 patients per group and no differences between groups; d) the number of trials with 50 patients per group and an effect size of 0.25 in favor of control.

The third sensitivity analyses will omit subsets of trials based on trial quality. We will first omit trials graded as 'B' for blinding from the comparison of acupuncture to sham. We will then repeat all analyses separately omitting trials that score fewer than 5 on the assessment of acupuncture adequacy.

Our final sensitivity analysis will be to add the results of studies for which we did not receive individual patient level data. We will calculate an estimate of the difference between groups and the standard error thereof from published summary data. As we will have individual patient data from all of the large trials of acupuncture, we do not believe that any remaining trials will have an important weight in the meta-analysis.

#### Additional analyses

##### Variation in the effects of acupuncture by indication

To determine whether the effects of acupuncture vary by indication, we will combine data into a single model predicting the standardized primary endpoint using baseline score, trial, indication, treatment and treatment by indication interaction, and test the hypothesis that all interaction terms are equal to zero. This analysis will be conducted separately for acupuncture vs. sham and acupuncture vs. no acupuncture control.

##### Analysis of acupuncture characteristics

In these analyses, we will examine whether characteristics of the acupuncture treatment affect outcome. We will examine the following trial level characteristics: number of sessions; frequency of sessions; duration of sessions; point prescription (fixed needle formula/flexible formula/individualized); stimulation (none/manual/electrical/both); prescription (local points only/distal points only/both local and distal points); 'style' of acupuncture ('Western'/traditional Chinese/other); 'De qi' needle sensation, whether felt by practitioner or patient (sought/not sought); number of needles used; use of adjunctive therapies such as moxibustion (yes or no); acupuncture-specific patient practitioner interactions (yes or no); minimum years of practice as practitioner requirement to be participating in trial. Patient levels characteristics to be examined are as follows: sex of acupuncturist; age of acupuncturist; medical training (MD, other professional qualification); length of acupuncturists' training in hours; years of experience as acupuncturist. Each characteristic will be examined separately using an interaction analysis of the complete data set, with trial entered as a fixed effect. For example, to determine whether number of sessions influences the effect of acupuncture our model would be:

Standardized final score=β1.Standardized baseline score+β2.Acupuncture+β3.Number ofsessions+β4.Number of sessions×Acupuncture

Here 'acupuncture' is coded 0 or 1 for control and treatment groups respectively. A significance test for β_4 _would then be used to test the hypothesis that the effects of acupuncture depend on the number of treatment sessions. If indicated, we may include several characteristics of acupuncture in a multivariable analysis although it is possible that there may be collinearity (e.g. trials that allowed individualized treatment also allowed acupuncture specific patient practitioner interactions).

##### Analysis of the effects of sham control

In this analysis, we will address the question of whether different types of sham control have different effects. In the first analysis, we will compare sham to control using the methods described above for the comparison of acupuncture to sham. We will report the effect sizes for the three types of sham - penetrating needle, non-penetrating needle, non-needle - and compare formally by meta-regression using two dummy variables - needle (coded 1 for penetrating needle and non-penetrating needle and 0 for non-needle) and penetration (coded 1 for penetrating needle, 0 otherwise). We will conduct three exploratory analyses: following the work of Sanchez Aranjo[38], we will use an additional variable for site of needling coded 1 for any needle penetration in the same dermatome as the active points and 0 otherwise; we will also add a variable for depth of insertion of the sham (superficial or not); for non-penetrating needles, we will determine whether devices placed at true acupuncture points have different effects to those placed away from true points. Indication (shoulder pain, headache, osteoarthritis, musculoskeletal pain) will be entered as a covariate in these analyses.

##### Time course of acupuncture effects

To estimate the time course of acupuncture effects, we will use a longitudinal model, that is, we will take into account the correlation between an individual patient's scores. We will use pain score as the dependent variable, baseline score, time and indication (headache, back or neck pain, shoulder pain, osteoarthritis), trial and acupuncture as predictors. In this analysis, we will use all time points in a trial, not just the time point specified as primary by the study authors. We will compare both acupuncture to sham and acupuncture to no acupuncture control in this analysis. The analysis will be repeated separately for each indication. The *xtgee *command will be used in Stata. The results will be presented numerically and also graphed with standardized mean difference as the y-axis and time since randomization as the x-axis. This will give a visual representation of how the benefits of acupuncture, if any, change over time. It will also allow estimation of effect size at specific times after randomization. As a secondary analysis, we will explore whether differences in the time course of acupuncture effects depends on the duration of acupuncture treatment. For example, the effects of acupuncture at six months may differ for a trial in which treatment is completed within 6 weeks, and in which treatment effects have 20 weeks to taper off, than for a trial in which patients are treated for 4 or 5 months. In this secondary analysis, time from last acupuncture treatment will be added as a covariate.

##### Analysis of acupuncture responders

Some acupuncturists claim that a subset of patients, who are difficult to identify in advance, are acupuncture 'responders' and have exceptional improvements after acupuncture therapy. We will test this hypothesis by thinking in terms of statistical distributions: if a subset of patients has unusual improvements after acupuncture, we would expect the distribution of change scores to have greater absolute skew in the acupuncture group than in controls. Accordingly, we will calculate the skew in standardized change scores for acupuncture and control groups separately, compute the difference in the skewness statistic and estimate a standard error for this difference by bootstrapping. The difference in skew and standard error of each trial will then be meta-analyzed. We will initially compare acupuncture to combined sham and no acupuncture control groups; subsequent analysis will compare acupuncture to sham and no acupuncture control separately. We will also calculate a p value for each trial separately by permutation methods: we will randomly permute the indicator for group, calculate the difference in skew, repeat 10,000 times and calculate a p value for each trial as the proportion of times that the resulting statistic is equal to or greater in absolute size than the skewness calculated for the original data.

##### Analysis of differences between practitioners

We will examine heterogeneity between practitioners, that is, whether some acupuncturists get better results than others. In our initial analysis, we will estimate heterogeneity for each trial separately by running the principal analysis - analysis of covariance with baseline score and randomization strata as covariates - separately for each practitioner. The resulting coefficients and standard errors will be combined and heterogeneity statistics calculated[37]. For a meta-analysis of differences between practitioners, each practitioner's coefficient will be standardized by calculating the absolute difference from the group level estimate of acupuncture effects. For example, if in a trial, the mean difference between groups calculated in the primary analysis was 0.5 standard deviations, and the difference for one practitioner was 0.4 standard deviations, the value 0.1 would be entered into the meta-analysis.

##### Analysis of predictors of acupuncture effect

In these analyses, we will examine effect modifiers, defined as characteristics of a patient, known before treatment, that influence the degree of benefit experienced by that patient. An example is Her-2/neu overexpression modification of Herceptin effect in a patient with advanced breast cancer: the likelihood that a patient's tumor will respond to Herceptin depends on whether or not the tumor overexpresses Her-2/neu. Effect modifiers will be examined by an interaction analysis of the complete data set, with trial entered as a fixed effect. For example, to determine whether age influences the effect of acupuncture, our model would be: Standardized final score = β_1_. Standardized baseline score + β_2_. Acupuncture + β_3_. Age + β_4_. Age × Acupuncture. A significance test for β_4 _would then be used to test the hypothesis that the effects of acupuncture are different in older compared to younger patients. We will test the effects of the following variables: age, sex, disease duration, baseline severity, baseline psychological distress. We may also examine indication-specific effect modifiers, for example, pain radiating to the legs in back pain trials: this will depend on the availability of these data in the meta-analyzed trials.

##### Implementation of the meta-analysis

A series of steps will be taken before each meta-analysis is conducted.

1. *Initial data collection*. A list of trials potentially eligible for the meta-analysis will be created. For example, a two-arm trial of acupuncture versus sham for the treatment of low back pain will be included for a meta-analysis of acupuncture versus sham for musculoskeletal pain, but not in a meta-analysis of acupuncture versus usual care, or one focusing on neck pain. If there are fewer than three eligible trials, meta-analysis will not be attempted and steps 2 - 5 will be omitted.

2. *Consultation*. The statistical center will prepare the following and make available to each collaborator:

a. A full copy of the main publication for each trial

b. Annotated statistical code that will describe, step-by-step, the statistical analyses to be undertaken. This document will be written broadly so that it will apply to analyses of all pain types, endpoints, time points and so on. In order to illustrate how the code will work, collaborators will also receive a print-out of the results from running the code on a data set where the indicator for treatment has been randomly permuted.

3. *Comment period*. Each collaborator will then have a four week period in which to:

a. Agree to the inclusion of all trials in the meta-analysis

b. State that one or more trials should not be included, giving a reason for each

c. Ask for more time.

4. *Collation of data*. The results of the collaborator comments will be combined.

a. If no collaborator recommends exclusion of any trial, we will move to step 5

b. If there is consensus that one or more trials should be excluded, the reasons will be documented and we will move to step 5

c. If there is disagreement over the inclusion of one or more trials, a description of the disagreement - the trials concerned, and the reasons why some collaborators are suggesting exclusion - will be distributed to all collaborators

d. A vote on each trial will then be taken

5. *Decision on the trials to be included in the meta-analysis*.

a. A final list of trials will be generated.

b. If there are only three trials, the statistical code for the meta-analysis will be run up to the point where meta-analytic weights are calculated. If the fixed effect weight for a single trial is 75% or greater, the meta-analysis will be halted and no further action will be taken; otherwise, the meta-analysis will proceed as in point 6.

c. If a meta-analysis includes more than three trials and one trial has a weight of 75% or greater, an adjustment will be made to weighting factors. The standard error of the largest trial will be increased by 25% and the weights recalculated. If the weight of the largest trial is less than 75%, we will proceed to step 6. Otherwise, the standard error of the largest trial will be increased by 33% and the weights recalculated. If the weight of the largest trial is less than 75%, we will proceed to step 6. Otherwise, meta-analysis will be halted.

6. *Final implementation of the meta-analysis*.

a. The remaining statistical code will be run and results will be written up by the statistics team and distributed to all collaborators.

## Discussion

We believe that the findings of the Acupuncture Trialists' Collaboration will have important implications for both clinical practice and research. Individual patient data meta-analysis of high quality trials will provide the most reliable basis for treatment decisions about acupuncture. Analyses as to the impact of different sham techniques, styles of acupuncture or frequency and duration of treatment sessions will no doubt guide future clinical trials of acupuncture.

Above all, however, we hope that our approach can serve as a model for future studies in acupuncture and other complementary therapies. In the Acupuncture Trialists' Collaboration, a group of trialists, statisticians and other researchers has come together to share raw data and develop, in partnership, a set of research questions and associated analytic strategies. We strongly believe that it is only by breaking down the oppositional culture of competing trialists, and sharing data in a robust scientific collaboration, that we can best translate clinical trial findings into patient benefit.

## Competing interests

The authors declare that they have no competing interests.

## Authors' contributions

The study was conceived by AV, GL, CW, and KL. AV was responsible for the overall study design with input from AC for the statistical analysis, AM for the systematic review, GL and HM with respect to acupuncture analyses, NV, CW, KS and KL with respect to clinical trial methodology and meta-analysis. The manuscript was written by AV and AM. All authors gave comments on early drafts and approved the final version of the manuscript.

## Supplementary Material

Additional file 1 **Outcome Measure Preferences**. This document is to guide the selection of endpoints to be included in the meta analyses. Endpoints are classified by domain (e.g. WOMAC pain is in the "pain intensity" domain; days of headache is in the "pain frequency" domain). This document specifies which endpoint should be chosen if a trial has data on more than one endpoint per domain. In general, we have given preference to measures that are specific to pain types, then to the most widely used measures. Click here for file
