# Supplementary material for: Individual patient data meta-analysis of acupuncture for chronic pain: protocol of the Acupuncture Trialists' Collaboration
Source: Trials. 2010 Sep 28;11:90. doi: 10.1186/1745-6215-11-90 (PMC2955653; doi:10.1186/1745-6215-11-90)
Supplement: Additional file 1 — Outcome Measure Preferences. This document is to guide the selection of endpoints to be included in the meta analyses. Endpoints are classified by domain (e.g. WOMAC pain is in the "pain intensity" domain; days of headache is in the "pain frequency" domain). This document specifies which endpoint should be chosen if a trial has data on more than one endpoint per domain. In general, we have given preference to measures that are specific to pain types, then to the most widely used measures. [file 1745-6215-11-90-S1.DOC]

# Additional File 1

**Outcome Measure Preferences**

This document is to guide the selection of endpoints to be included in the meta analyses. Endpoints are classified by domain (e.g. WOMAC pain is in the "pain intensity" domain; days of headache is in the "pain frequency" domain). This document specifies which endpoint should be chosen if a trial has data on more than one endpoint per domain. In general, we have given preference to measures that are specific to pain types, then to the most widely used measures.

**Pain Frequency**

**Headache**

**For trials only with migraine patients**

1. days with migraine
2. days with migraine symptoms
3. Have you had migraine during the last 4 weeks? (1=yes/0=no)
4. migraine attacks
5. List as below

**For trials including non-migraine headache**

1. days with moderate/strong headache
2. days with headache
3. days with strong headache
4. total headache hours
5. days with headache from other causes
6. During the past 4 weeks have you experienced TTH? (1=yes, 0=no)
7. Do you have other pain than TTH? (1=Yes/0=no)
8. days with impaired activity
9. During the past 4 weeks have you had migraine as well (as TTH)? (1=yes, 0=no))
10. Do you have other pain in addition to migraine headache (1=yes/0 = no)
11. days with at least 1 accompanying symptom
12. days with a minimum of 1 accompanying symptom

**Low back pain**

- - 1. Days with pain in the past month (diary 31 days max)

**Knee and/or hip osteoarthritis**

1. days with pain in the last week (diary 7 days max)

**Shoulder**

1. Pain persistent at time of assessment
2. New episode of pain at time of assessment

**Pain Intensity**

1. Diary scores (if pain diaries use more than one measure of pain, then preference is as below)
2. VAS and NRS will be combined by multiplication (NRS*10 = VAS)
   1. Average pain measured by the VAS or NRS will be first choice
   2. If a trial measures:
      1. If worst, day and night scores - we will average day and night scores
      2. If day and night scores are given – we will take the average
      3. If good and bad knee scores are given - we will average the two scores
      4. If average, highest intensity, lowest and current are given – we will use average
      5. If pain severity in the past week, unpleasantness in the past week, and severity of main problem are given - we will use severity in the past week
3. WOMAC pain
4. SES pain affective/sensoric
5. Arthritis Self-Efficacy scale - pain subscale
6. SPADI Shoulder pain and disability index PAIN COMPONENT (0-50)
7. Quality of life (SF36): bodily pain
8. McGill pain questionnaire
9. Global headache severity
10. Constant Murley Pain (CMS) component
11. Von Korff Q1-3 (pain intensity)

**Functional Impairment**

1. WOMAC function
2. Constant Murley (CMS) activity component
3. Disability Oswestry
4. Disability: Roland Disability Scale (RMDQ)
5. Back function score (FFbH-R 0-100%)
6. SPADI Shoulder pain and disability index DISABILITY (0-80)
7. Days with impairment due to pain
   1. Days with limited function because of pain (180 days max)
   2. Days unable to do household work
   3. Days with inability to work due to pain
8. Disability: Pain Disability Index (PDI)
9. Von Korff Q5-7 (impairment)
10. Walking ability

**Combined pain and functional impairment**

1. WOMAC index/combined
2. Neck pain and disability score
3. Constant Murley Scale (Constant Murley) total
4. Northwick Park Neck (NPQ - measures Pain and disability)
5. OARSI-OMERACT Responder (pain structure function scale)
6. SPADI Shoulder pain and disability index GLOBAL (0-130)
7. Oxford score

**Mental QoL**

1. Quality of life (SF36): mental component score
2. Average of Quality of life (SF36): social functioning, role emotional, and mental health components
3. SF12 - Mental Health Summary Score
4. Average of Profile of the Quality of Life in the Chronically Ill (PQLC) psychological, positive mood, negative mood, social functioning, and social wellbeing components
5. Average of HAD Anxiety and HAD Depression components
6. Worry about neck pain compared to worry felt at previous questionnaire
7. Other depression scores
8. Other anxiety scores

**Physical QoL**

1. Quality of life (SF36): physical component score
2. Average of Quality of life (SF36): physical activity and role physical
3. Profile of the Quality of Life in the Chronically Ill (PQLC) physical
4. SF12 - Physical Health Summary Score

**Overall QoL**

1. SF36 total score (1-100)
2. Quality of life (SF36): general health perception
3. Quality of Life COOP/WONCA CHARTS (30-0) lower scores mean higher QOL
4. Quality of life (SF36): vitality
5. Euro QOL (not reported in paper)

**Range of motion/stiffness**

1. WOMAC stiffness
2. Average of Constant Murley Scale (CMS) range of motion, abduction, flexion, external rotation, internal rotation, strength
3. Average of active and passive cervical mobility
4. Range of Movement

**Health change**

1. Self-evaluated improvement NRS 0-10
2. Assessment of treatment effect (1-5)
3. Global assessment of change (1-5)
4. Quality of life (SF36): health change over the past year 0-10
5. Globally improved, judged by clinician (yes/no)
6. Physician evaluated improvement NRS 0-10

**Satisfaction**

1. Satisfaction with treatment outcome
2. Satisfaction with care
3. Satisfaction with information

**Medication**

1. Days taking medication
2. Medication quantification scale
3. Consumption of drugs (none, below, equal, above, extra than prescribed)
4. Any use of pain medication
5. Amount of trial drug taken (diclofenac) measured by weight of remaining pills
6. Other
